# The BCL2L11 deletion polymorphism is not associated with imatinib resistance in chronic myeloid leukemia patients: meta-analysis

**DOI:** 10.18632/oncotarget.21154

**Published:** 2017-09-22

**Authors:** Jinyun Xu, Jiaowei Gu, Yan Zhao, Huihua Meng, Li’an Du, Ruibo Zhang, Hao Jiang, Jianming Luo

**Affiliations:** ^1^ Department of Pediatrics, The First Affiliated Hospital of Guangxi Medical University, Nanning, Guangxi, China; ^2^ Department of Pediatrics, Taihe Hospital, Hubei University of Medicine, Shiyan, Hubei, China

**Keywords:** BIM, chronic myeloid leukemia, tyrosine kinase inhibitor, genetic polymorphism, drug resistance

## Abstract

A common deletion polymorphism of the gene Bcl-2 like protein 11 (BCL2L11, BIM) has been reported to cause tyrosine kinase inhibitors (TKIs) resistance in several malignant tumors. However, the conclusions were not consistent in chronic myeloid leukemia (CML) individuals. In order to obtain a reliable conclusion, we systematically searched PubMed, Embase, Web of Science, Chinese Biomedical Database, and China National Knowledge Infrastructure and performed the meta-analysis. Six published articles contain 760 East Asian patients were identified from these electronic databases. The methodological quality of one included trial was high, and the others were moderate. Meta-analysis showed that the rate of TKI resistance between the BIM deletion and wild-type group were no statistical significance (OR = 1.24, 95% CI 0.79–1.95). In conclusion, BIM deletion may not a predictor of TKI resistance in CML individuals in East Asia.

## INTRODUCTION

Chronic myeloid leukemia (CML) is a malignancy of hematology caused by the reciprocal chromosomal translocation t (9; 22) and constitutively active BCR-ABL [1, 2], which affects about one individual per 100,000 population per year [3–6]. Usually, the clinical course of CML is characteristically triphasic: Chronic phase, acceleration and blast crisis, and symptoms are controlled more easily in chronic phase [4]. Fortunately, most patients tend to be diagnosed in the chronic phase [7, 8], and the treatment to this phase has improved over the past decades [1, 9–14].

Since 1996, Druker et al. [2] reported a novel compound (imatinib) for the effect of the tyrosine kinase inhibitor (TKI) on CML cell lines. In 2001, imatinib was approved for the treatment of CML in phase 2 studies [15]. In recent years, imatinib was used as the front-line treatment of chronic phase CML patients all over the world [1, 6, 14, 16–24]. However, the rate of imatinib resistance have been reported about 20%, and even more if added imatinib intolerance [22–24]. Thus, further in-depth analysis of the mechanisms of imatinib resistance in CML patients are necessary.

Ng et al. [25] recently identified a common intron 2 deletion polymorphism in the gene encoding Bcl-2 like 11 (BCL2L11, BIM). The BIM deletion polymorphism appeared to occur at a frequency of 12.3% individuals only in East Asia, and showed an inferior response to tyrosine kinas inhibitors (TKIs) when compared to those without the deletion in CML patients [25]. In addition, this common BIM deletion may predict relapse after TKI discontinuation [26]. However, other studies suggested that BIM deletion was not significantly associated with the TKI efficacy for CML patients [27, 28]. Accordingly, we decided to conduct the meta-analysis of currently available studies to assess the relationship between BIM deletion and imatinib resistance in CML patients.

## RESULTS

### Literature search and study selection

**Figure 1:**
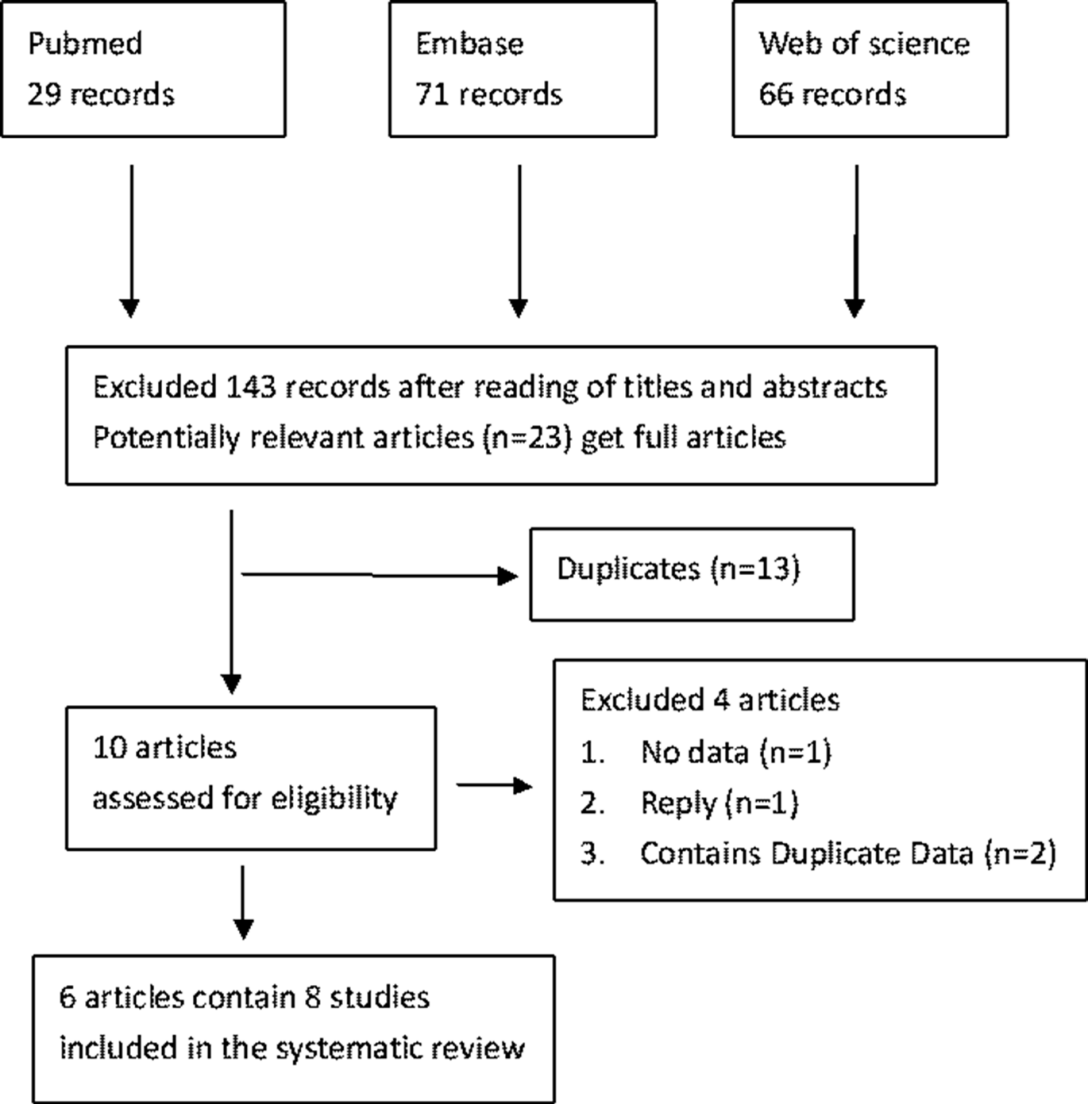
Flowchart of the study identification

We identified 28 records from PubMed, 71 records from Embase, 66 records from Web of Science and none from CBD and CNKI. After reviewing the titles and abstracts, we obtained 23 possible involvement articles and get these full-text articles for further evaluation. In these 23 possible articles from three different data-bases, there were 13 duplicates. Two articles contain duplicate data, one study without enough data and one reply were excluded from the last 10 articles. Ultimately, 6 articles [25, 27–31] including 8 studies were enrolled in the study. No additional trials were identified by checking the reference lists. The study selected process was depicted in Figure 1.

### Characteristics and quality of the studies

There were 6 articles [25, 27–31] involving 760 patients included in this meta-analysis. All of the patients were treated with imatinib as the front-line treatment except that one study switching to nilotinib after more than 18 months’ treatment of imatinib [30]. All of the included patients were reported in chronic phase with positive BCR-ABL fusion gene and the primary outcome was on the basis of the European LeukemiaNet (ELN) criteria.

Only 1 study-data was obtained prospectively from multi-center [27], and others were retrospective analysis. One trial received the quality score of 4 [30] and two received 5 [29, 31], illustrated that the methodological quality was low. The characteristics and quality score of each study were presented in Table 1.

**Table 1:** Characteristics of the included researches

Study ID	Country	Sample size	BIM deletion	Bcr-abl	Drugs dose	Sensitive/ Resistant	Study design	Data sources	standard	outcome	NOS score
Ng 2012 A	Singapore Malaysia	138	15	+	Imatinib 0.4 g/d	69/ 69	retrospective	Multi-center	ELN	Sensitive Resistant	9
Ng 2012 B	Japan	65	12	+	Imatinib 0.4 g/d	25/ 40	retrospective	Multi-center	ELN	Sensitive Resistant	9
Katagiri 2013 A	Japan	37	2	+	Imatinib NA	20/ 17	retrospective	Single center	ELN	Sustained or fluctuating CMR > 24 month	5
Katagiri 2013 B	Japan	13	3	+	Imatinib NA	5/ 8	retrospective	Single center	ELN	Maintained CMR > 12 OR < 12 month after stop imatinib	5
Shinohara 2013	Japan	144	15	+	Imatinib 0.4 g/d	72/ 72	prospective	Multi-center	ELN	CMR	6
Chen 2014	China	220	30	+	Imatinib	140/ 80	retrospective	Multi-center	ELN	Sensitive Resistant	7
Miyamura 2016	Japan	40	3	+	Nilotinib after Imatinib 0.4 g/d 18 month	27/ 13	retrospective	Multi-center	ELN	MMR at 24 months	4
Than 2016	Japan	103	15	+	Imatinib	89/ 14	retrospective	Single center	ELN	10-year OS	5

CMR, complete molecular response; +, positive; d, day; NA, not available; ELN, European LeukemiaNet; MMR, major molecular response; OS, overall survival.

**Figure 2:**
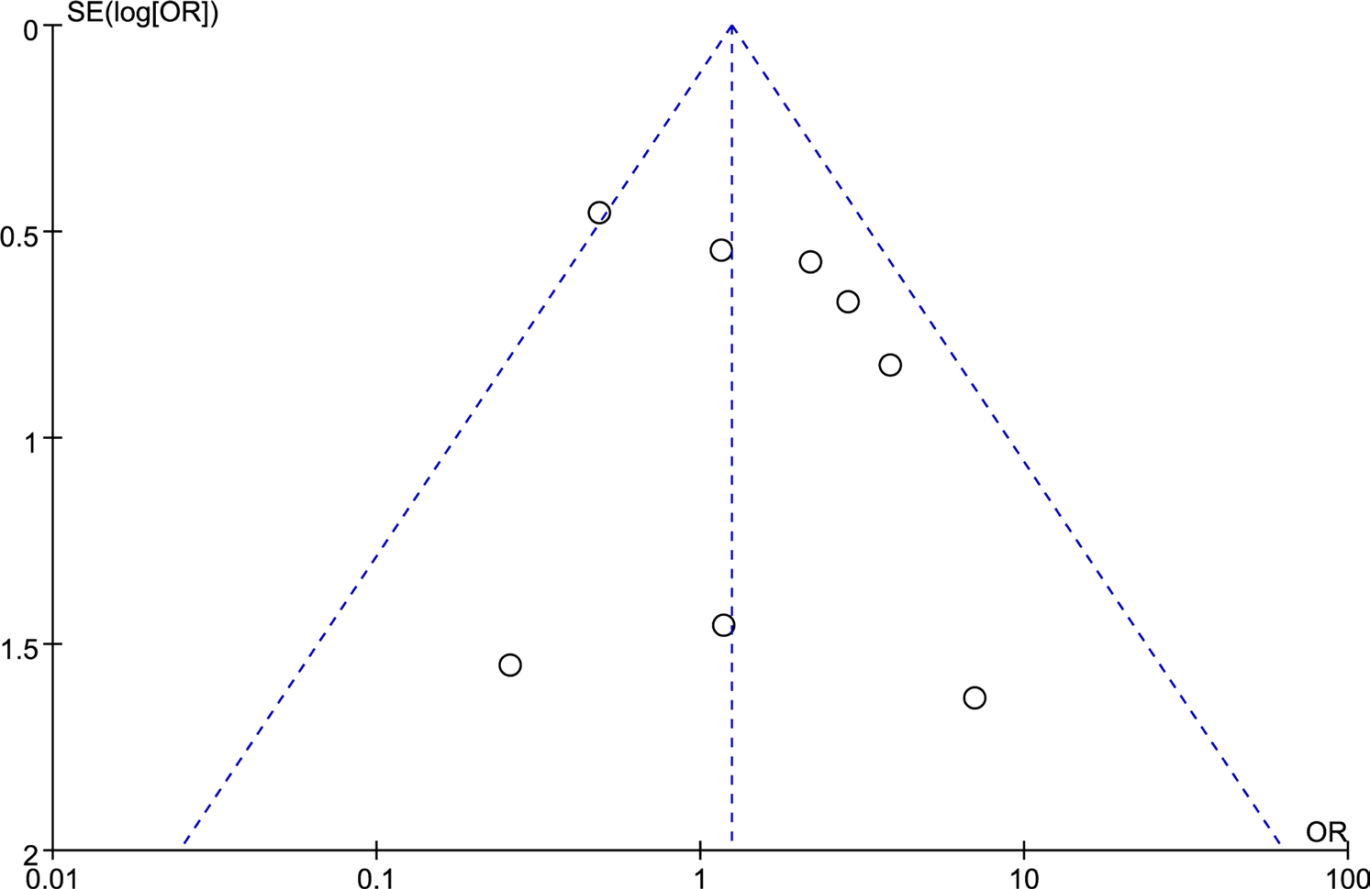
Funnel plot of TKI-resistance between the BIM deletion and wild-type

There was no significant asymmetry in funnel plots for the outcomes between the BIM deletion and wild genotype by TKI treatment (Figure 2).

### Data synthesis

**Figure 3:**
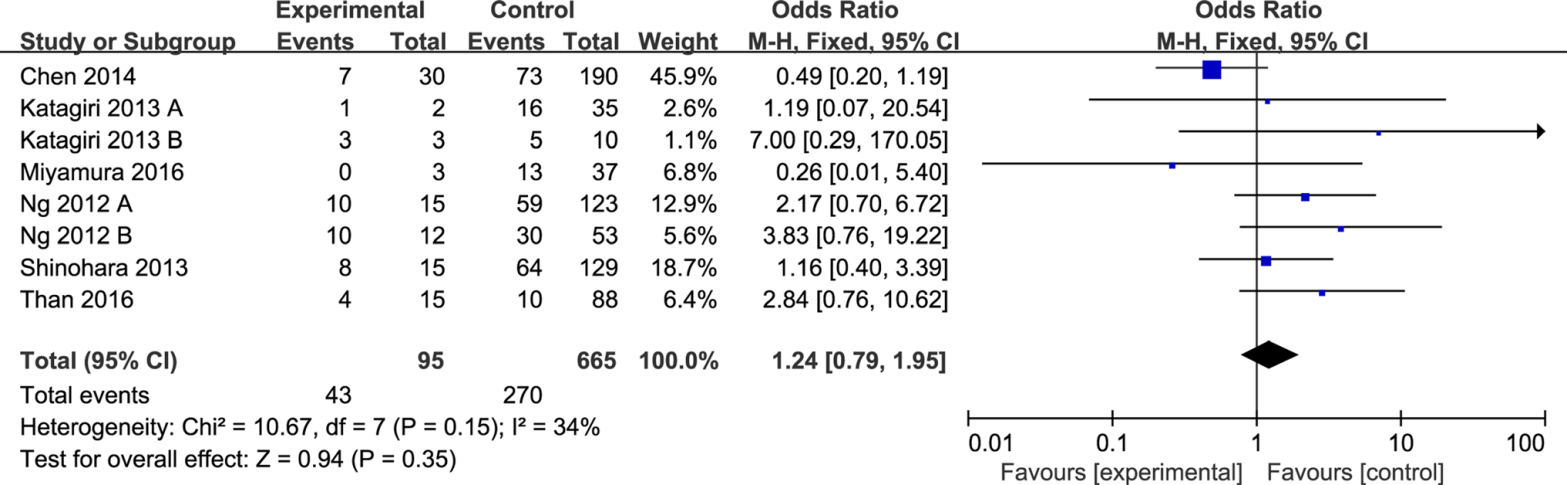
Meta-analysis of the association between the BIM deletion polymorphism and imatinib-resistance in CML patients

According to the ELN criteria [24], we preformed meta-analysis to synthesize these results through TKI-resistant rate (Table 2 and Figure 3). The rate of TKI-resistance in CML patients who harbored BIM deletion or not were no statistical significance (OR = 1.24, 95% CI 0.79–1.95). There was no statistical significance between included studies in heterogeneity (*I*^2^ = 34%, *P* = 0.15). There were similar results whether we calculate OR or RR, and no matter we used the fixed or random model (Table 2).

**Table 2:** The results of Meta-analysis between BIM deletion type and wild type in resistance to TKI

Effect measures	Effect model	I^2^ (*P*)	95% CI	*P*
OR	fixed	34% (*P*= 0.15)	1.24 (0.79–1.95)	0.35
OR	random		1.42 (0.73–2.73)	0.30
RR	fixed	37% (*P* = 0.14)	1.12 (0.89–1.41)	0.34
RR	random		1.26 (0.95–1.68)	0.11

OR: odds ratio, RR: risk ratio, CI: confidence intervals

**Figure 4:**
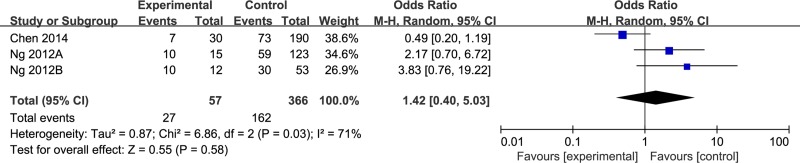
Subgroup analysis of two articles which defined the results in a same manner

There were two articles contain 3 studies which defined the results in the same manner on the basis of the ELN [25, 28]. Then, we performed subgroup analysis using these data (Figure 4). There was significant heterogeneity in this subgroup, we performed meta-analysis using random-effects model. There was no statistical significance between the two groups at the rate of TKI-resistance.

## DISCUSSION

It is well known that the gene BIM encodes a Bcl-2 homology domain 3 (BH3) only protein, which is a pro-apoptotic member of B-cell lymphoma 2 (Bcl-2) family [32, 33]. BIM could induce hematologic cancer cell death through apoptotic pathway [32]. Previous studies have shown that imatinib activated pro-apoptotic BH3-only protein BIM, which is regarded as a major role in imatinib induced apoptosis of the BCR-ABL1 positive CML cells [34, 35]. However, a common 2903 bp intron deletion polymorphism of BIM leads to the preferential generation lack the BH3 domain and it may correlated with inferior response to TKI in CML patients [25]. Notably, there were three studies reported the contradictory results [27, 28, 30]. Hence, we used data from published studies and performed this meta analysis.

In this study, we found that BIM intron 2 deletion polymorphism was not associated with TKI resistance in CML patients (OR = 1.24, 95% CI 0.79–1.95). In subgroup analysis, we combined data from two studies [25, 28] and also found similar result (OR = 1.42, 95% CI 0.40–5.03). These results suggesting that BIM deletion polymorphism may be not associated with clinical efficacy of TKI therapy in CML individuals in East-Asian.

Recent studies showed that dasatinib [11] and nilotinib [12, 13] was superior to imatinib in both major molecular response and complete cytogenetic response. Even in patients with CML who are resistant to imatinib therapy, dasatinib may induces notable response [1, 10]. When patients with BIM polymorphisms experience a suboptimal response to imatinib, switching to nilotinib would benefit them [30]. In summary, if BIM deletion was associated with imatinib-resistance, the common BIM deletion would become a symbol of excluded imatinib for treating CML in East-Asian. However, the results of the systematic review proved that this common BIM deletion were not related to clinical relevance of imatinib-resistance. We suggested that this common BIM deletion should not used as a symbol of discontinuation of imatinib or switching imatinib to other TKIs.

Nowadays, TKI targeting BCR-ABL1 is the standard of care for patients with CML in chronic phase [9, 17, 18, 30, 36, 37]. Response during TKI therapy is the most important prognostic factor for long-term outcome in CML. Since there are not enough evidences suggesting that BIM deletion polymorphism is related to TKI-resistance in CML patients, we propose the common BIM deletion should not serve as a biomarker for determining the prognosis in CML patients with the treatment of TKIs.

There is only one study reported a subset of non high-risk CML patiets and found that BIM deletion was associated with inferior 10 years over sur*vivo*rs [31]. Nevertheless, the result is from a retrospective study and the included number of individuals are small so that further investigation is warranted.

There are several reasons affecting the quality of evidence. Firstly, this systematic review included only published trails. Secondly, the quality score of each trials could be graded as moderate except one [25]. Thirdly, the results between studies were defined inconsistently. Fourthly, there were no randomized controlled trails.

In conclusion, this systematic review and meta analysis shows that the common BIM deletion is not associated with TKI-resistance in CML patients in East Asian. Through this systematic review, we also suggest that the common BIM deletion should not be served as an indicator to discontinue imatinib or switching imatinib to other TKIs. However, further prospective studies included large trails which defined the results with widely accepted criteria are essential.

## MATERIALS AND METHODS

We used different search strategies and searched more electronic databases than those by Ying et al. [38]. The recommendations of the preferred reporting items for systematic review and meta-analysis were the main methods used for this study [39].

### Literature search

We comprehensively searched PubMed, Embase, Web of Science, Chinese Biomedical Database (CBD), and China National Knowledge Infrastructure (CNKI) from inception until May 2017. Keywords and search terms were as following, “BIM OR BCL2L11 OR Bcl-2 like 11” AND “deletion OR polymorphism” AND “CML OR chronic myeloid leukemia OR chronic leukemia”. Next, we searched reference lists of all included articles for additional relevant trial. There was no language restrictions.

### Inclusion and exclusion criteria

We included relevant articles if they fulfilled the following eligibility criteria. (1) Retrospective or prospective studies which investigated the association between BIM deletion polymorphism and TKI efficacy of CML patient. (2) There were sufficient data concerning outcomes. (3) patients were receiving TKIs therapy. (4) response assessments were according to the international guidelines. Conversely, review, meta-analysis and case report were excluded. If there were any studies with duplicated data, we included only one which with larger sample size and more information.

### Literature screening and data extraction

Two review authors (Jinyun and Jiaowei) assessed all of the studies searched from the databases through titles and abstracts. We obtained the full articles if some papers may satisfied our criteria and then reviewed the literatures carefully to decided whether included or not. At last, reference list of eligible studies were identified. If there were any disagreements between the two reviewers about study inclusion, we resolved them by discussion in our study group.

The author Jinyun extracted study details from the included trials. These data were verified by another two authors (Yan Zhao and Jiaowei).

### Assessment of risk of bias

Two reviewers (Jinyun and Jiaowei) independently assessed the risk of bias in all included studies according to an assessment tool of the Newcastle-Ottawa Scale (NOS) that is recommended by the Cochrane Collaboration [40, 41]. The score of the methodological quality of all included studies were range from 0 to 9, which the higher score represented the higher quality. Disagreements were resolved by discussion in our group.

### Statistical meta analysis

RevMan 5.3 was used for this meta-analysis. For Dichotomous data, we calculated odds ratios (OR) or risk ratios (RR) corresponding 95% confidence intervals (CI). The heterogeneity was evaluated by the Q test and I^2^ statistic. The I^2^ statistic ranges from 0% to 100%, a value of 0% indicated no observed heterogeneity and larger values show increasing heterogeneity [42]. If the I^2^ < 50% and *P* value ≥ 0.1, we considered heterogeneity was no significance and used the fixed effects model for analysis. Otherwise, the potentially inconsistency among all included trails were analyzed carefully, if the heterogeneity was not excluded we used the random-effects model.
